# Pregnancy Monitoring in Potiskum and Neighboring Communities of Yobe State, Nigeria: Challenges and Maternal Health Implications in Rural Settings

**DOI:** 10.7759/cureus.82381

**Published:** 2025-04-16

**Authors:** Olajide J Olagunju, Egbo Ben, Olayinka Olagunju, Oluwadamilola G Majolagbe, Olagoke O Osanyinlusi, Titilade O Adewoye, Omolola F Atoyebi

**Affiliations:** 1 Division of Infectious Disease, Case Western Reserve University School of Medicine, Cleveland, USA; 2 Biostatistics and Epidemiology, East Tennessee State University, Johnson City, USA; 3 Department of Radiology, Potiskum Medical Center, Potiskum, NGA; 4 Department of Zoology, Obafemi Awolowo University, Ile-Ife, NGA; 5 Nursing, University of Ottawa, Overland Park, USA; 6 Department of Anesthesiology, Washington University School of Medicine in St. Louis, St. Louis, USA; 7 Sustainability and Environmental Management, Coventry University, Coventry, GBR; 8 Nursing, Barnes Jewish Hospital, St. Louis, USA

**Keywords:** health systems, maternal health, rural nigeria, sociocultural barriers, yobe state

## Abstract

Background

Pregnancy monitoring through timely antenatal care (ANC) is a cornerstone of maternal and neonatal health. However, in rural and underserved communities of northeastern Nigeria, numerous structural and sociocultural barriers continue to limit access to essential monitoring services, contributing to poor pregnancy outcomes. This study investigates the barriers influencing pregnancy monitoring in Potiskum and neighboring communities in Yobe State, Nigeria.

Methods

A retrospective cross-sectional study was conducted using electronic health records from Potiskum Medical Center between 2018 and 2023. Data from 14,846 pregnant women were analyzed. Adequate pregnancy monitoring was defined as having eight ANC visits and at least one ultrasound before 24 weeks of gestation. Bivariate and multivariate logistic regression analyses were performed to identify predictors of inadequate monitoring.

Results

Women aged >35 had the highest odds of inadequate pregnancy monitoring (adjusted odds ratio (AOR): 4.66, 95% CI: 3.94-4.66, p < 0.001), with elevated risks also observed in the 26-30 (AOR: 2.62, 95% CI: 2.32-2.81) and 30-35 age groups (AOR: 2.78, 95% CI: 1.42-3.92; both p < 0.001). Low education (AOR: 2.62, 95% CI: 2.11-2.72, p < 0.01), unemployment (AOR: 2.32, 95% CI: 2.12-2.85, p = 0.001), and lack of health insurance (AOR: 2.69, 95% CI: 1.99-2.73, p < 0.0001) were strong predictors, while tertiary level of education reduced the risk (AOR: 0.42, 95% CI: 0.24-0.48, p < 0.0001). Limited decision-making autonomy (AOR: 3.74, 95% CI: 3.22-4.03, p < 0.0001) increased the odds of inadequate monitoring, whereas absence of negative healthcare experiences (AOR: 0.12, 95% CI: 0.08-0.41) and non-adherence to traditional beliefs (AOR: 0.14, 95% CI: 0.10-0.22; both p < 0.0001) were protective factors.

Conclusion

A combination of socioeconomic disadvantage, cultural norms, and health system shortcomings drives inadequate pregnancy monitoring in rural Yobe State. Interventions that promote women’s autonomy, expand insurance coverage, address traditional misconceptions, and improve patient experiences within healthcare facilities are urgently needed to improve ANC utilization and maternal health outcomes in underserved Nigerian communities.

## Introduction

Pregnancy monitoring provides a structured approach to tracking fetal development, assessing maternal well-being, and identifying potential complications early. Ensuring timely medical interventions helps prevent adverse pregnancy outcomes, reduce maternal and neonatal complications, and improve overall survival rates. Through regular antenatal visits, ultrasound assessments, and routine screenings, pregnancy monitoring ensures that mothers receive the necessary care to promote safe deliveries and healthier postnatal outcomes [1]. However, in rural and underserved regions, access to essential pregnancy monitoring services remains severely limited, increasing the risk of maternal and neonatal morbidity and mortality [2,3]. Potiskum and its neighboring communities in Yobe State, Nigeria, exemplify this reality, where challenges such as limited healthcare infrastructure, socioeconomic barriers, cultural beliefs, and inadequate access to diagnostic tools like ultrasound hinder effective pregnancy monitoring.

Nigeria continues to rank among the countries with the highest maternal mortality rates worldwide, maintaining one of the world’s highest maternal mortality rates at over 800 deaths per 100,000 live births [4]. Disproportionately, rural communities bear the greatest burden due to limited antenatal care (ANC) coverage, inadequate healthcare infrastructure, and delays in seeking medical attention [2]. Despite clear evidence that routine pregnancy monitoring, including regular antenatal visits, ultrasound scans, and proactive management of high-risk pregnancies, dramatically reduces maternal and neonatal complications, many pregnant women in these underserved areas struggle to access these critical, life-saving services [1,5]. Financial constraints, geographic isolation, cultural beliefs, and systemic healthcare gaps create formidable barriers, leaving many women vulnerable to preventable complications that endanger both their lives and those of their unborn children. Urgent action is needed to bridge these gaps and ensure that all pregnant women, regardless of location, receive the essential care they deserve.

By conducting a thorough analysis of the systemic, economic, and cultural obstacles that restrict access to maternal healthcare, this research will uncover the underlying causes of inadequate pregnancy monitoring and assess its widespread impact on maternal and neonatal health outcomes. These barriers, ranging from limited healthcare infrastructure and financial constraints to deeply ingrained cultural beliefs and geographical inaccessibility, continue to hinder timely and effective maternal care in rural communities. Using a comprehensive data-driven approach, this study will generate robust, evidence-based insights to guide the development of targeted strategies and policy recommendations to strengthen maternal health services and bridge the healthcare disparity in rural settings. Addressing these barriers is not merely an urgent necessity; it is an indispensable step toward closing the maternal healthcare gap, eliminating preventable pregnancy-related complications, and significantly improving survival rates for mothers and newborns across Nigeria’s rural communities.

Aims and objectives

This study aims to identify and understand the systemic, economic, and cultural barriers hindering adequate pregnancy monitoring in rural Nigeria. 

The objectives of this study include (1) To determine the prevalence of adequate and inadequate pregnancy monitoring among women residing in Potiskum and surrounding villages in Yobe State, Nigeria; (2) To examine the influence of sociodemographic, economic, systemic, and cultural factors on pregnancy monitoring practices in rural Nigerian communities; (3) To generate evidence-based recommendations aimed at improving access to maternal healthcare and reducing disparities in rural Nigeria.

## Materials and methods

Study design

This is a retrospective cross-sectional study that examined five years (2018-2023) of data from the electronic health records (EHRs) of Potiskum Medical Center (PMC), a leading healthcare facility in northeastern Nigeria, serving Potiskum and neighboring rural communities in Yobe State, Nigeria. The study evaluates pregnancy monitoring practices, focusing on maternal healthcare utilization, antenatal care visit patterns, and the barriers affecting access to essential maternal health services.

Study settings

Potiskum Medical Center, the fastest-growing private healthcare facility in the region, serves as a referral center for over 500,000 individuals across Potiskum and rural communities in Yobe State, Nigeria. Potiskum is a rapidly growing town and the largest commercial center in Yobe State, Nigeria, and drives economic activity through agriculture, livestock trade, and transport networks. Despite its growth, healthcare access remains limited, especially in surrounding rural communities where maternal health services are inadequate. The Hausa, Fulani, and Kanuri populations predominantly inhabit the area, with cultural traditions influencing healthcare decisions. Many residents depend on both public and private healthcare facilities, but long travel distances, financial constraints, and cultural beliefs continue to hinder pregnancy monitoring and maternal healthcare utilization.

Study population

The study included women who presented in pregnancy at Potiskum Medical Center during the study period (January 1, 2018, to December 31, 2023). Inclusion criteria were women who had at least one documented antenatal visit at Potiskum Medical Center during the study period, women with complete records on antenatal visits, gestational age at first visit, medical history, and pregnancy outcomes. Women residing in Potiskum or nearby rural areas within Yobe State who received antenatal care at the facility. Exclusion criteria were women who visited the facility for general medical issues but did not receive antenatal care, women who had an initial antenatal care visit at Potiskum Medical Center but completed it at another facility, and women who traveled from other states or regions for emergency care but were not part of the local population 

Data preparation

The selected variables underwent a thorough review to ensure accuracy, consistency, and relevance to the study objectives. To enhance data integrity, variables were re-coded and standardized to align with established research methodologies and best practices in maternal health studies. This process ensured that the data structure was suitable for meaningful analysis and comparison within the study framework. The outcome of interest (pregnancy monitoring) was re-coded as a binary variable. To align with the 2016 WHO recommendation for a positive pregnancy experience, adequate monitoring was defined as at least eight antenatal visits and one or more ultrasound scan before 24 weeks gestation; inadequate monitoring as fewer than eight antenatal visits and no ultrasound scan before 24 weeks gestation [6].

Independent variables (barriers to pregnancy monitoring) include maternal factors such as age, which was re-coded from detailed age to the following age categories: 16-20, 21-26, 26-30, 31-35, and above 35 years; level of education, which was simplified into four categories: no formal education, primary education, secondary education, and tertiary education; parity, which was simplified into first pregnancy or one or more previous pregnancy. Economic factors such as employment status, which was described as employed or unemployed; household income level, which was simplified into three levels: low income (<₦50,000/month), middle income (₦50,000-250,000/month), and high income (₦250,000/month); and health insurance coverage, “Yes” or “No”. Healthcare system factors such as distance to PMC, which was simplified into two categories: <5km (kilometers) or >5km; waiting time at the health facility, also simplified into two categories: short (less than one hour) or long (one hour or longer) and previous negative experience with healthcare services, “Yes” or “No”. Sociocultural factors such as who makes healthcare decisions, simplified as self or husband/his family, and the Influence of traditional beliefs on antenatal care are described as Yes or No.

Statistical analysis

A total of 14,846 records were included in this study. Descriptive statistics were carried out to understand the characteristics of the study population. Simple logistic regression analyses were carried out to examine the independent association between the dependent variable (outcome variable) and independent variables. The backward selection method included variables for the multivariate logistic regression model, which assessed the combined association between pregnancy monitoring and barriers to pregnancy monitoring. Variables demonstrating statistical significance in the simple logistic regression models were included in the multivariate model to evaluate their combined effects. Statistical significance was set at p < 0.05, and all analyses were performed using the Statistical Analysis System (SAS) version 9.4 for Windows (SAS Institute Inc., Cary, NC, USA).

## Results

Table 1 presents the descriptive characteristics of the 14,846 records included in the study. Most participants were between the ages of 21 and 30 years (63.21%), with the 26-30 age group being the most represented (34.39%). Educational attainment varied, with 33.98% having completed secondary education, while 22.83% had no formal education. Two-thirds (66.83%) of the women had experienced more than one pregnancy, and a significant majority (75.20%) were unemployed. Health insurance coverage was reported by 42.15%, while 57.85% lacked insurance.

**Table 1:** Descriptive Statistics of the Study Population in Potiskum and Neighboring Communities, Yobe State, Nigeria ANC: antenatal care; PMC: Potiskum Medical Center

Variables	Categories	Frequency (N=14,846)	Percentage (%)
	16-20	1,514	10.19
	21-25	4,280	28.82
Age	26-30	5,107	34.39
	30-35	2,988	20.19
	>35	947	6.41
	No formal education	3,390	22.83
Level of education	Primary education	3,772	25.40
	Secondary education	5,044	33.98
	Tertiary Education	2,640	17.79
Parity	First pregnancy	4,925	33.17
	> One previous pregnancy	9.921	66.83
Employment status	Employed	3,682	24.80
	Unemployed	11,164	75.20
Health Insurance Coverage	Yes	6,258	42.15
	No	8,588	57.85
Distance to PMC	< 5km	6,382	42.99
	> 5km	8,464	57.01
Waiting time	Short	13,587	91.52
	Long	1,259	8.48
Previous negative experiences with healthcare services	Yes	5,488	36.97
	No	9,358	63.03
Who makes healthcare decisions?	Self	2,237	15.07
	Husband/his family	12,609	84.93
Traditional beliefs about ANC	Yes	5,743	38.70
	No	9,101	61.30

Regarding healthcare access, 57.01% lived more than 5 km from PMC, and 91.52% reported short waiting times during visits. Notably, 36.97% of participants had experienced negative encounters with healthcare services in the past. Most healthcare decisions were made by the husband or his family (84.93%), and 38.70% of respondents endorsed traditional beliefs regarding ANC. These characteristics highlight a population shaped by limited educational attainment, socioeconomic constraints, and cultural influences that may affect pregnancy monitoring behaviors and maternal health outcomes.

Table 2 presents the results of a bivariate logistic regression analysis, which we used to assess how each selected predictor variable independently influences the likelihood of adequate pregnancy monitoring among women in Potiskum and neighboring rural communities of Yobe State, Nigeria. By examining these associations individually, we identified several potential barriers that may significantly impact whether pregnant women receive the necessary level of monitoring throughout their pregnancy.

**Table 2:** Bivariate Logistic Regression Analysis of Independent Associations Between Predictor Variables and Pregnancy Monitoring ANC: antenatal care; PMC: Potiskum Medical Center; OR: odds ratio; CI: confidence interval

Variables	Categories	OR (CI)	Wald Test (χ²)	P Value
	16-20	Ref		
	21-25	1.22 (1.04-1.68)	2.64	<0.001
Age	26-30	2.37 (1.85-2.49)	129.62	<0.001
	30-35	2.92 (1.63-3.48)	464.18	<0.001
	>35	4.32 (3.77-4.92)	935.22	<0.001
	No formal education	Ref		
Level of education	Primary education	2.81 (2.24-2.92)	233.39	0.002
	Secondary education	1.64 (1.22-2.87)	22.64	0.0012
	Tertiary Education	0.48 (0.31-0.57)	67.52	<0.0001
Parity	First pregnancy	Ref		
	> One previous pregnancy	0.92 (0.74-1.84)	0.15	0.060
Employment status	Employed	Ref		
	Unemployed	1.72 (1.21-1.92)	26.93	0.002
Health Insurance Coverage	Yes	Ref		
	No	2.24 (1.84-2.46)	91.29	<0.0001
Distance to PMC	< 5km	Ref		
	> 5km	0.62 (0.54-0.76)	0.30	0.86
Waiting time	Short	Ref		
	Long	0.47 (0.39-0.98)	3.51	0.092
Previous negative experiences with healthcare services	Yes	Ref		
	No	0.18 (0.09-0.25)	124.45	<0.0001
Who makes healthcare decisions?	Self	Ref		
	Husband/his family	4.22 (3.88-4.42)	304.88	<0.0001
Traditional beliefs about ANC	Yes	Ref		
	No	0.10 (0.02-0.21)	222.36	<0.0001

Table 3 presents the results of the multivariate logistic regression analysis examining factors independently associated with inadequate pregnancy monitoring. The multivariable logistic regression model demonstrated strong predictive performance, with the variables showing statistically significant adjusted odds ratios and high Wald χ² statistics. Multicollinearity was assessed using the Variance Inflation Factor (VIF). All VIF values were below 5, indicating no serious multicollinearity among predictors. Therefore, the estimates from the logistic regression model are considered stable and interpretable.

**Table 3:** Multivariate Logistic Regression to Access the Combined Effect of the Barriers Influencing Pregnancy Monitoring in Potiskum and Neighboring Communities of Yobe State, Nigeria ANC: antenatal care; AOR: adjusted odds ratio; CI: confidence interval

Variables	Categories	AOR (CI)	Wald Test (χ²)	P Value
	16-20	Ref		
	21-25	1.28 (1.24-1.98)	4.28	<0.001
Age	26-30	2.62 (2.32-2.81)	176.52	<0.001
	30-35	2.78 (1.42-3.92)	388.25	<0.001
	>35	4.66 (3.94-4.66)	1292.10	<0.001
	No formal education	Ref		
Level of education	Primary education	2.62 (2.11-2.72)	221.06	<0.001
	Secondary education	1.24 (1.01-2.63)	178	<0.001
	Tertiary Education	0.42 (0.24-0.48)	168	<0.0001
Employment status	Employed	Ref		
	Unemployed	2.32 (2.12-2.85)	253.92	0.001
Health Insurance Coverage	Yes	Ref		
	No	2.69 (1.99-2.73)	158.44	<0.0001
Previous negative experiences with healthcare services	Yes	Ref		
	No	0.12 (0.08-0.41)	58.46	<0.0001
Who makes healthcare decisions?	Self	Ref		
	Husband/his family	3.74 (3.22-4.03)	629.76	<0.0001
Traditional beliefs about ANC	Yes	Ref		
	No	0.14 (0.10-0.22)	151.81	<0.0001

Age showed a strong and progressive association, with women aged >35 years significantly more likely to experience inadequate monitoring compared to those aged 16-20 years (AOR: 4.66, 95% CI: 3.94-4.66, p < 0.001). Similar trends were observed in the 26-30 and 30-35 age groups (AOR: 2.62 and 2.78, respectively; p < 0.001). The educational level also had a significant effect: women with primary education were more likely to experience inadequate monitoring (AOR: 2.62, 95% CI: 2.11-2.72), while those with tertiary education were less likely to do so (AOR: 0.42, 95% CI: 0.24-0.48, p < 0.0001). Unemployed women had significantly higher odds of inadequate monitoring than those who were employed (AOR: 2.32, 95% CI: 2.12-2.85, p = 0.001). Lack of health insurance was also a strong barrier (AOR: 2.69, 95% CI: 1.99-2.73, p < 0.0001). Interestingly, women who had not reported previous negative experiences with healthcare services were less likely to have inadequate monitoring (AOR: 0.12, 95% CI: 0.08-0.41, p < 0.0001). Furthermore, those whose husbands or family members made healthcare decisions had significantly higher odds of inadequate monitoring compared to women who made decisions themselves (AOR: 3.74, 95% CI: 3.22-4.03, p < 0.0001). Lastly, women who did not hold traditional beliefs about ANC were significantly less likely to experience inadequate monitoring (AOR: 0.14, 95% CI: 0.10-0.22, p < 0.0001).

## Discussion

In this study, we uncovered several key sociodemographic, systemic, and cultural barriers that significantly contribute to inadequate pregnancy monitoring among women in Potiskum and neighboring rural communities in Yobe State, Nigeria. Our analysis revealed that these barriers do not operate in isolation but interact in complex ways that hinder timely and consistent pregnancy monitoring. By shedding light on these multifaceted challenges, our findings emphasize the urgent need for targeted, context-specific interventions aimed at improving maternal health service access and uptake in rural and underserved populations.

Age emerged as a significant barrier to adequate pregnancy monitoring. Women aged above 35 years demonstrated nearly five times higher odds of receiving inadequate monitoring compared to those aged 16-20 years. This age-related disparity likely reflects a pattern in which older women, drawing on previous childbirth experiences, may underestimate the need for adequate pregnancy monitoring, making them present late for booking in the hospital. Tesfaye M et al. similarly reported this tendency in their study on delayed ANC initiation in selected public health institutions in Southwest Ethiopia, where older pregnant women were less likely to have adequate pregnancy monitoring [7]. Our findings also align with those of Tumwizere G et al., who, in a national study conducted in Uganda, observed that younger women were more likely to utilize ANC services than their older counterparts [8]. In contrast, younger women may be more receptive to health promotion messages out of curiosity or may experience stronger encouragement from family members and the community to attend ANC, contributing to better monitoring outcomes in this age group.

Education significantly influenced the likelihood of adequate pregnancy monitoring. Women with no formal education or only primary education faced a substantially higher risk of inadequate monitoring, whereas those with tertiary education were considerably more likely to receive appropriate care. This trend reinforces the well-established link between educational attainment and maternal health outcomes. Educated women are more likely to understand the benefits of ANC, recognize early signs of complications, and actively seek timely and consistent care. Furthermore, education equips women with the confidence and skills needed to navigate healthcare systems, communicate effectively with providers, and advocate for their health needs. These findings align with a growing body of literature demonstrating that improving female education is a key driver of better maternal and child health outcomes in low-resource settings [1,9-13].

Unemployment significantly increased the risk of inadequate pregnancy monitoring. Unemployed women faced more than twice the odds of receiving insufficient monitoring compared to their employed counterparts. This finding is consistent with the findings of other studies that have largely described the negative impact of unemployment on women of reproductive age in healthcare utilization. Mobolaji Victoria Adejoorin et al., in their study of the utilization of maternal health facilities and rural women’s well-being in Nigeria, agreed that unemployed women, especially in Northeastern Nigeria, where we conducted our study, are less likely to utilize maternal healthcare facilities [14]. Also, Aklilu Habte et al., in their study of the predictors of maternal health services uptake in the West African region, described unemployment as a significant predictor of maternal health services [15]. Other studies also mentioned unemployment as a significant factor in adequate pregnancy monitoring [16,17]. This association likely reflects the financial barriers and socioeconomic vulnerabilities that limit access to maternal healthcare services. Without a stable income, many women may be unable to afford transportation, healthcare costs, or other indirect expenses associated with antenatal care, ultimately contributing to poor utilization of essential services.

Similarly, women without health insurance faced significantly higher odds of receiving inadequate pregnancy monitoring, further highlighting the critical role that financial accessibility plays in shaping maternal health-seeking behavior. This finding is consistent with the findings of other studies within and outside Nigeria [18-21]. It reinforces the urgent need to strengthen and expand community-based health insurance schemes that reduce out-of-pocket costs and improve access to essential maternal services. In addition, policymakers and stakeholders must prioritize economic empowerment initiatives that enhance women’s financial autonomy, particularly in rural areas where socioeconomic vulnerability is often more pronounced. Ensuring that women can afford and access care is essential for improving ANC uptake and achieving equitable maternal health outcomes.

Household decision-making structures strongly influenced pregnancy monitoring. Women whose husbands or their family members controlled healthcare decisions were nearly four times more likely to receive inadequate pregnancy monitoring compared to those who exercised autonomy over their health choices. This is consistent with other studies that have found that women do better when they can make healthcare decisions. Chukwuechefulam Kingsley Imo, in his study of the Influence of women's decision-making autonomy on antenatal care utilization and institutional delivery services in Nigeria, concluded that women's autonomy promotes adequate pregnancy monitoring, especially in Nigeria [22]. Also, a study conducted in Bangladesh by Md Badsha Alam et al. concluded that women’s participation in healthcare decisions enables better utilization of maternal healthcare [23]. This finding underscores the enduring impact of patriarchal systems, which often diminish women's agency and create significant barriers to accessing timely and adequate maternal health services. When women lack the authority to make decisions about their care, they are less likely to seek preventive services, attend regular antenatal visits, or respond promptly to complications. Addressing these deep-rooted gender dynamics is essential for improving maternal health outcomes, particularly in rural and traditional settings.

Traditional beliefs surrounding pregnancy and ANC significantly contributed to inadequate pregnancy monitoring. Women who held culturally rooted misconceptions about pregnancy were far less likely to attend regular ANC visits or engage fully with maternal health services. In rural Yobe State, Nigeria, traditional beliefs significantly influence ANC utilization. Many communities view pregnancy as a natural process that requires no medical intervention unless complications arise, leading to delayed or missed ANC visits. Women often rely on traditional birth attendants due to cultural familiarity, spiritual trust, and fear that frequent hospital visits may attract evil spirits or result in unnecessary medical procedures. The stigma associated with seeking frequent care further hinders access. Additionally, the use of herbal remedies, fatalistic views about divine control over pregnancy outcomes, and cultural restrictions on movement during pregnancy all contribute to low engagement with formal healthcare services. This finding aligns with other research showing that such beliefs often discourage early or consistent ANC utilization [24-26]. To address these challenges, public health programs must implement targeted community education campaigns that respectfully challenge harmful norms and promote evidence-based maternal care.

Interestingly, women who did not report previous negative experiences, such as inattentive or incompetent care, discrimination, stigmatization, financial exploitation, and disrespectful or abusive treatment with healthcare services, were significantly less likely to receive inadequate pregnancy monitoring. This finding highlights the critical but often underappreciated role of healthcare quality and patient satisfaction in promoting sustained engagement with maternal services. When women encounter respectful treatment, timely care, and clear communication, they are more likely to develop trust in the health system and return for continued pregnancy monitoring. Onyeajam DJ et al., in their study of antenatal care satisfaction in a developing country, carried out in Nigeria, discussed extensively the positive impact of modifiable factors such as empathy and a non-discriminating environment in adequate pregnancy monitoring [27]. A study conducted in Adamawa State, Nigeria, by Hammanjoda K et al., described the need for continuous quality improvement of services in healthcare facilities to facilitate adequate pregnancy monitoring [28]. Finally, multiple other studies also emphasized the benefit of quality improvement in pregnancy monitoring [29-31]. Conversely, experiences marked by disrespect or dismissive attitudes can discourage women from seeking further care, even when needed. To improve maternal health outcomes, healthcare facilities must prioritize respectful maternity care, streamline service delivery, and foster patient-centered communication that reinforces women’s confidence in the system.

Strengths and limitations

This study offers several notable strengths. With a large sample size of 14,846 records collected over five years from Potiskum Medical Center, the findings are statistically robust and reflective of real-world maternal healthcare utilization in rural northeastern Nigeria. The use of electronic health records minimizes recall bias and enhances data reliability. Moreover, the study’s comprehensive approach, analyzing sociodemographic, economic, systemic, and cultural barriers, provides a nuanced understanding of the factors affecting pregnancy monitoring. However, some limitations should be acknowledged. As a single-center retrospective study, the findings may not be generalizable to other regions with differing healthcare systems or cultural dynamics.

## Conclusions

This study highlights the complex interplay of sociodemographic, economic, cultural, and systemic factors that contribute to inadequate pregnancy monitoring in Potiskum and surrounding rural communities in Yobe State, Nigeria. Key predictors such as low educational attainment, unemployment, lack of health insurance, and older maternal age significantly increased the risk of inadequate pregnancy monitoring. Cultural beliefs, limited autonomy in healthcare decision-making, and prior negative experiences with health services further compounded the problem, illustrating how deeply entrenched social norms and service delivery gaps jointly shape maternal health outcomes in underserved settings.

To improve maternal health uptake and reduce disparities, a multifaceted approach is essential. Interventions should prioritize empowering women to make health-related decisions, engaging communities through culturally sensitive education, and addressing systemic shortcomings in care delivery. Strengthening health insurance access, improving service quality, and fostering respectful, patient-centered care environments can build trust and encourage continued engagement with maternal health services. These actions are critical to advancing maternal health equity and achieving sustainable improvements in pregnancy outcomes across rural Nigeria.

## References

[1] Olagunju OJ, Egbo B, Osanyinlusi OO, Olagunju OE, Olorunmolu SE (2025). Assessing the role of ultrasound scanning in improving pregnancy outcomes in Potiskum and neighboring rural communities in Yobe State, Nigeria. Cureus.

[2] Adewuyi EO, Auta A, Adewuyi MI, Philip AA, Olutuase V, Zhao Y, Khanal V (2024). Antenatal care utilisation and receipt of its components in Nigeria: assessing disparities between rural and urban areas-a nationwide population-based study. PLoS One.

[3] Ojemeiri KA, Akintayo AO, Obada AA, Uchendu CE (2025). Maternal health status in Dikwa Local Government Area of Borno State, Nigeria. Konfrontasi.

[4] (2019). Maternal health in Nigeria: generating information for action. https://www.who.int/news/item/25-06-2019-maternal-health-in-nigeria-generating-information-for-action.

[5] Tukur J, Lavin T, Adanikin A (2022). Quality and outcomes of maternal and perinatal care for 76,563 pregnancies reported in a nationwide network of Nigerian referral-level hospitals. EClinicalMedicine.

[6] (2016). WHO Recommendations on Antenatal Care for a Positive Pregnancy Experience. https://www.who.int/publications/who-guidelines.

[7] Tesfaye M, Dessie Y, Demena M, Yosef T (2021). Late antenatal care initiation and its contributors among pregnant women at selected public health institutions in Southwest Ethiopia. Pan Afr Med J.

[8] Tumwizere G, K Mbonye M, Ndugga P (2024). Determinants of late antenatal care attendance among high parity women in Uganda: analysis of the 2016 Uganda demographic and health survey. BMC Pregnancy Childbirth.

[9] Babalola S, Fatusi A (2009). Determinants of use of maternal health services in Nigeria--looking beyond individual and household factors. BMC Pregnancy Childbirth.

[10] Adedokun ST, Uthman OA, Bisiriyu LA (2023). Determinants of partial and adequate maternal health services utilization in Nigeria: analysis of cross-sectional survey. BMC Pregnancy Childbirth.

[11] Amwonya D, Kigosa N, Kizza J (2022). Female education and maternal health care utilization: evidence from Uganda. Reprod Health.

[12] Muyunda B, Makasa M, Jacobs C, Musonda P, Michelo C (2016). Higher educational attainment associated with optimal antenatal care visits among childbearing women in Zambia. Front Public Health.

[13] Okedo-Alex IN, Akamike IC, Ezeanosike OB, Uneke CJ (2019). Determinants of antenatal care utilisation in sub-Saharan Africa: a systematic review. BMJ Open.

[14] Adejoorin MV, Salman KK, Adenegan KO, Obi-Egbedi O, Dairo MD, Omotayo AO (2024). Utilization of maternal health facilities and rural women's well-being: towards the attainment of sustainable development goals. Health Econ Rev.

[15] Habte A, Hailegebreal S, Simegn AE (2024). Predictors of maternal health services uptake in West African region: a multilevel multinomial regression analysis of demographic health survey reports. Reprod Health.

[16] Nwosu CO, Ataguba JE (2019). Socioeconomic inequalities in maternal health service utilisation: a case of antenatal care in Nigeria using a decomposition approach. BMC Public Health.

[17] Kitole FA, Shahid M, Bashir S (2025). Antenatal healthcare utilization and maternal health outcomes in rural African setting: evidence from Tanzania. Glob Soc Welf.

[18] Esan OT, Adeomi AA, Afolabi OT (2023). Health insurance coverage and access to maternal healthcare services by women of reproductive age in Nigeria: a cross-sectional study. BMJ Public Health.

[19] Yaya S, Da F, Wang R, Tang S, Ghose B (2019). Maternal healthcare insurance ownership and service utilisation in Ghana: Analysis of Ghana Demographic and Health Survey. PLoS One.

[20] Merga BT, Raru TB, Deressa A (2023). The effect of health insurance coverage on antenatal care utilizations in Ethiopia: evidence from national survey. Front Health Serv.

[21] Wang W, Temsah G, Mallick L (2017). The impact of health insurance on maternal health care utilization: evidence from Ghana, Indonesia and Rwanda. Health Policy Plan.

[22] Imo CK (2022). Influence of women's decision-making autonomy on antenatal care utilisation and institutional delivery services in Nigeria: evidence from the Nigeria Demographic and Health Survey 2018. BMC Pregnancy Childbirth.

[23] Alam MB, Khanam SJ, Kabir MA, Chowdhury AR, Hassen TA, Das S, Khan MN (2025). Effects of women’s participation in household decision making on skilled birth attendants supervised delivery in Bangladesh. Health Serv Insights.

[24] Igbokwe CC, Ihongo JT, Abugu LI, Iweama CN, Ugbelu JE (2024). Influence of cultural beliefs on the utilization of integrated maternal, newborn, and child health services in Benue State, Nigeria. Cureus.

[25] Opara UC, Iheanacho PN, Li H, Petrucka P (2024). Facilitating and limiting factors of cultural norms influencing use of maternal health services in primary health care facilities in Kogi State, Nigeria; a focused ethnographic research on Igala women. BMC Pregnancy Childbirth.

[26] Ikechukwu HU, Ofonime NU, Kofoworola O, Asukwo DE (2020). Influence of cultural and traditional beliefs on maternal and child health practices in rural and urban communities in Cross River State, Nigeria. Ann Med Res Pract.

[27] Onyeajam DJ, Xirasagar S, Khan MM, Hardin JW, Odutolu O (2018). Antenatal care satisfaction in a developing country: a cross-sectional study from Nigeria. BMC Public Health.

[28] Hammanjoda K, Singh AG (2024). The effect of healthcare service quality dimensions on patient satisfaction among primary care settings in Nigeria. Glob Soc Welf.

[29] Kagan I, Porat N, Barnoy S (2019). The quality and safety culture in general hospitals: patients', physicians' and nurses' evaluation of its effect on patient satisfaction. Int J Qual Health Care.

[30] Ope BW, Wasan T, Hirst JE, Mullins E, Norton R, Peden M (2025). Measurement, determinants and outcomes of maternal care satisfaction in Nigeria: a systematic review. BMJ Public Health.

[31] Guspianto G, Hubaybah H, Ningsih VR (2022). Quality of service and its effect on patient value, patient satisfaction, and revisit intention: investigation of the public health center in Jambi Province. Open Access Maced J Med Sci.

